# Evaluating soil metallic pollution and consequent human health hazards in the vicinity of an industrialized zone, case study of Mubarakeh steel complex, Iran

**DOI:** 10.1186/s40201-015-0231-x

**Published:** 2015-10-30

**Authors:** Zohreh Ghaemi, Abdolreza Karbassi, Faramarz Moattar, Amirhesam Hassani, Nematollah Khorasani

**Affiliations:** Department of Environmental Science, Graduate School of the Environment and Energy, Science and Research Branch, Islamic Azad University, Tehran, Iran; Graduate Faculty of Environment, University of Tehran, P.O. Box 14155-6135 Tehran, Iran

**Keywords:** Metal pollution, Soil, Mobility, Mubarakeh steel complex, Isfahan

## Abstract

**Background:**

Being established in 1988 in the vicinity of Isfahan city, Mubarakeh Steel complex has imposed adverse environmental and health effects within the area. The study area is covered by lots of farms through which major crops like wheat and rice are provided.

**Methods:**

Considering the imposed pollution load of the complex, the current study has monitored the concentration of metals Fe, Al, Cd, Cr, Ni, Pb, Cu, Zn, Mn, Co, Mo, As in 14 soil samples within the study area. Furthermore, human health hazards of mentioned metals due to consumption of domestic rice and wheat have also been evaluated through different scenarios. In order to evaluate the mobility of metals in soil samples the sequential chemical analysis is performed.

**Results:**

Regarding the accumulation of metals in loose phases the order of metals bioavailability risk level is estimated to be as follows:

Co > Cd > Mo > Ba > As > Pb > Mn > Cu > V > Zn > Cr > Ni

**Discussion:**

An index approach is also considered to evaluate the severity of metal contamination. Regarding geochemical accumulation index, only cadmium is detected to be in a moderately contaminated status while other metals declare an unpolluted condition. Index of pollution pays more attention to mobility potential of metals and accordingly detects metals Co, Mn, As, Pb, Cd, Ba and Mo to be in a moderately contaminated level. On the other hand, enrichment factor declares all toxic metals except for Co, Ba and V to be enriched.

**Conclusions:**

Considering human health hazard assessment, except for Fe, Ba, Cu and Zn, all metals intakes in different scenarios are considered as hazardous while their CDI values are much more than the respective oral reference doses.

## Introduction

Metal contaminated sites contain a potentially hazardous risk for human beings and the environment. The bioavailability of such pollution is sophisticatedly used as an indicator of potential risk [1, 2]. Metals may exist in various species in the environment, where they may be transformed from one form into another or exist in different forms simultaneously. Depending on environmental conditions, the chemical speciation of metals changes widely. Such differences in chemical speciation play a key role in forming the environmental fate, bioaccessibility and environmental risk of the metals.

Knowledge on metals bulk concentrations alone may not be sufficient enough to evaluate the adverse effects of contaminated soils while toxic metals are present in different chemical forms in soil (easily exchangeable ions, metal carbonates, oxides, sulfides, organometallic compounds, ions in crystal lattices of minerals, etc.), which determine their mobilization capacity and bioavailability [3–5].

Knowledge of metal’s speciation may help to evaluate the metal tendency to remain in soil or sediments and how easily it may be released into the water phase.

Sequential extraction procedures are among the most widely used methods through which several extractants are used consecutively to separate the portion of phases from the bulk metallic concentration in a soil sample.

A vast variety of extraction processes providing information about the strength of bonds between metal species and soil fractions have been used by different researchers [2, 6–8]. However, none has been fully accepted as a universal method to be applied for all types of soils metallic pollution studies. Such lack of integrity may result in an erroneous diagnosis in soils. The study of potential mobility involves summing up all reactions that are capable of mobilising metals [9].

**Fig. 1:**
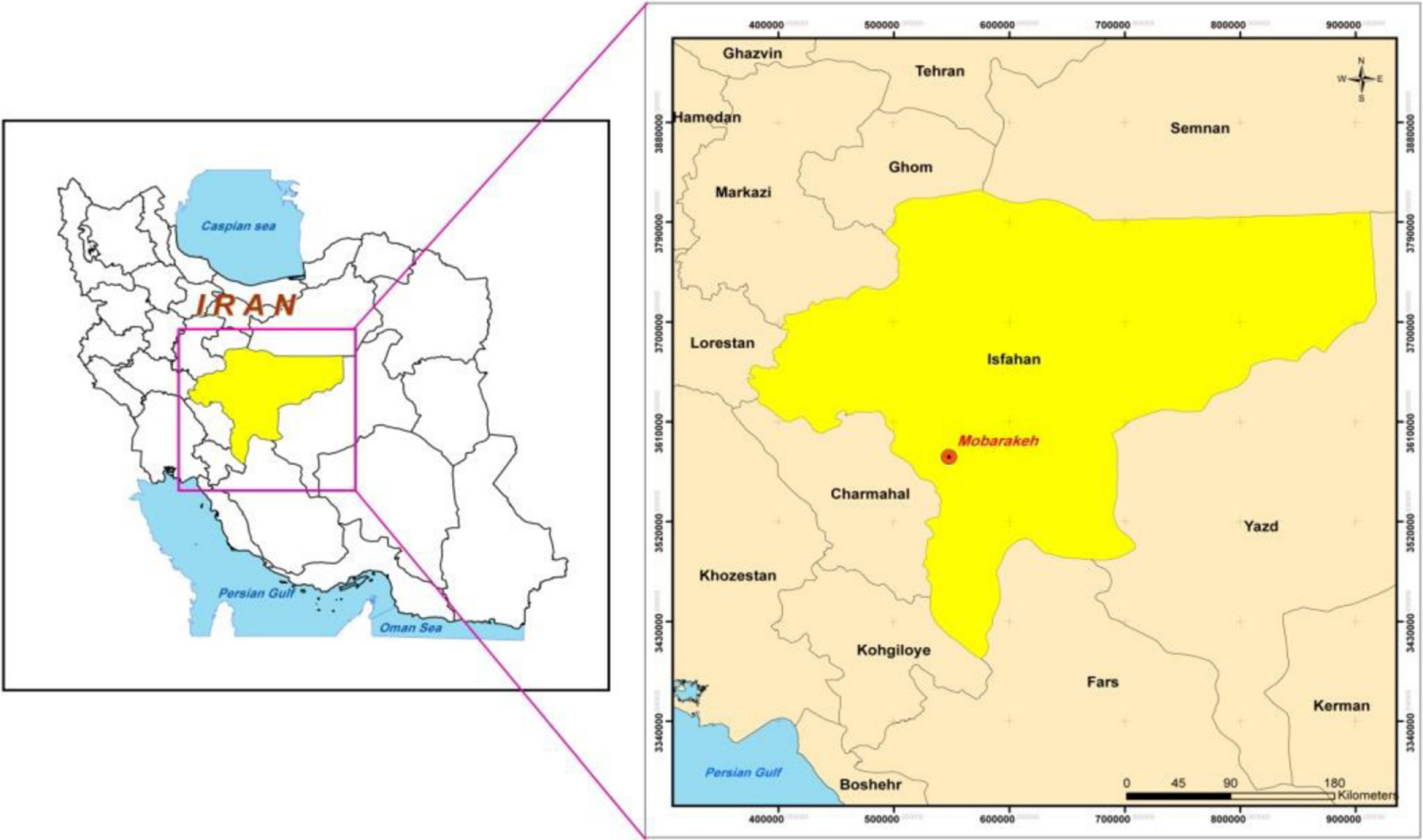
Study area within Iran and Isfahan province

Being established in 1988 in the vicinity of Mubarakeh county, Mubarakeh Steel complex has imposed adverse environmental and health effects within the area. A remarkable increase in the number of reported disease cases in Mubarakeh and Zarrinshahr in recent years may be attributed to the mentioned complex activities. The study area is covered by lots of farms through which major crops of Isfahan province are provided. The study area within Iran is demonstrated in Fig. 1.

Considering the imposed pollution load of the complex, the current study has monitored the concentration of metals iron, aluminum, cadmium, chromium, nickel, lead, copper, zinc, manganese, cobalt, molybdenum and arsenic in soil samples within the study area.

The current study presents not only the bulk concentration of eight metals (Fe, Al, Cd, Cr, Ni, Pb, Cu, Zn, Mn, Co, Mo, As) in superficial soil samples of Mubarakeh steel complex vicinity, but also the chemical partitioning of mentioned elements. The latter findings create a clear view of metals potential mobility and consequently the tendency to be absorbed by plants or animals within the bioaccessibility phenomenon. For this study, a modified sequential extraction procedure has been considered [10, 11].

## Materials and methods

**Fig. 2:**
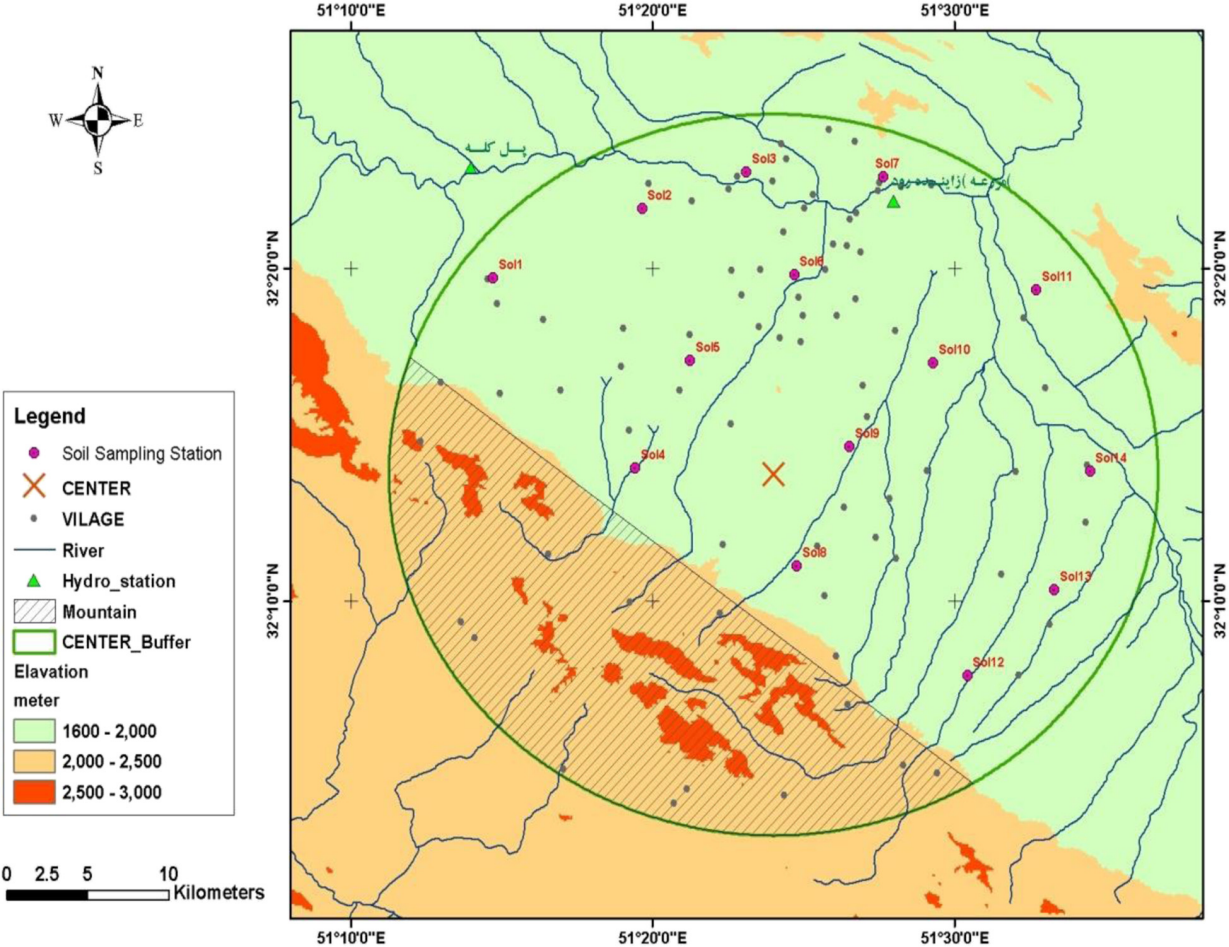
Layout of soil sampling stations within the study area

Soil samples were collected from 14 stations in the vicinity of Mubarakeh steel complex during the summer of 2012 using a Peterson grab sampler (Fig. 2). Spatial details of sampling locations are indicated in Table 1.

**Table 1:** Spatial characteristics of soil sampling stations

Station	UTM X	UTM Y
1	51.24535	32.32831
2	51.32745	32.36294
3	51.38493	32.3814
4	51.32361	32.23317
5	51.35387	32.28718
6	51.41157	32.33025
7	51.46045	32.37887
8	51.4129	32.18419
9	51.44207	32.24389
10	51.48821	32.28587
11	51.54518	32.32231
12	51.50707	32.12949
13	51.55509	32.17231
14	51.57471	32.23179

Soil samples, which were brown to grey in color, were air-dried and passed through a 63-μm mesh (equivalent to a No. 230 sieve, ASTM E-11). Being powdered using an agate mortar and pestle, about 0.5 g of the sample was placed in a Teflon beaker containing 10 mL aqua regia.

The mixture was heated until most of the liquid had evaporated, and allowed to cool before 5 mL of hydrogen fluoride (HF) were added. The samples were further cooled to room temperature before being filtered. The filtrates were transferred to 50 mL volumetric flasks and brought to volume with 1 N HCl [10]. Slightly modified from the old procedure, chemical partitioning was conducted in three sequential steps: (1) acetic acid 25 % v/v, (2) acetic acid 25 % v/v-0.1 M hydroxylamine hydrochloride and (3) 30 % H202 “extraction with 1 M ammonium acetate” [11]. To digest rice and wheat samples, 15 milliliters of the mixture HNO_3_:H_2_SO_4_:HClO_4_ 1:1:5 was added to 1 gram of powdered rice/wheat and heated to 80° centigrade. The mixtures were further cooled and filtered before injection process [12].

The analysis of metals in solutions was carried out by an inductively coupled plasma atomic emission spectrometer. Certified reference material (CRM 320) was used to check the accuracy of the analytical procedures. Replicate analysis of this CRM showed an acceptable accuracy, with recovery rates for metals between 92 % and 105 %. Furthermore, two samples were analyzed in triplicate to determine the precision of the analytical processes. The average values of the variation coefficients were estimated to be less than 10 % which may be considered acceptable for such studies [13].

To have an estimate of background status, according to soil samples collected from the vicinity of Mubarakeh steel, metals concentrations in shale have been considered. Such assumption is normally used as background values in sediment studies [13–16].

In order to determine the value of metal concentrations in different sedimentary phases, the modified sequential chemical partitioning method was considered [11].

To evaluate the severity of metallic pollution within the study area, geochemical accumulation index, enrichment factor and index of pollution were taken into consideration.

An index approach was used to assess the severity of metal contamination in the vicinity of Mubarakeh steel complex. Accordingly, the geochemical accumulation index was calculated using:

1 $$ \mathrm{Igeo} = \mathrm{Log}2\ \left[\mathrm{C}\mathrm{n}/\left(1.5 \ast \mathrm{B}\mathrm{n}\right)\right] $$

Where Igeo stands for geochemical accumulation index, Cn is the bulk metal concentration and Bn is the average metal concentration in shale [17, 18].

The enrichment factor (EF) values was calculated for metals using:

2 $$ \mathrm{E}\mathrm{F} = \left[\left(\mathrm{C}\mathrm{n}/\mathrm{C}\mathrm{F}\mathrm{e}\right)\ \mathrm{sample}\right]\ /\ \left[\left(\mathrm{C}\mathrm{n}/\mathrm{C}\mathrm{F}\mathrm{e}\right)\mathrm{crust}\right] $$

Where (Cn/CFe) sample indicates the ratio of the concentration of a specific element (Cn) to that of Fe (CFe) in soil sample and (Cn/CFe) crust is the same ratio in an unpolluted reference sample [19].

Finally, index of pollution which is a modification of Igeo is referred by the following formula:

3 $$ \mathrm{IPOLL} = \mathrm{Log}2\ \left[\mathrm{B}\mathrm{c}/\mathrm{L}\mathrm{p}\right] $$

The study area is a major agricultural site in the province and crops like wheat and rice are widely cultivated within the region. In order to evaluate the probable crops metal uptake, metals concentration in eight different rice and 15 different wheat samples were measured.

To evaluate the hazard level threatening local habitants due to consumption of rice and wheat products, nutritional pattern and physiological characteristics of residents should be taken into consideration. Accordingly six different scenarios are defined (Tables 2 and 3).

**Table 2:** Details of rice consumption scenarios within the study area

Scenario No.	1	2	3	4	5	6
Body weight (kg)	70	70	70	15	15	15
Daily rice consumption (gr)	100	250	400	100	250	400

**Table 3:** Details of wheat consumption scenarios within the study area

Scenario No.	1	2	3	4	5	6
Body weight (kg)	70	70	70	15	15	15
Daily wheat consumption (gr)	150	300	450	150	300	450

Chronic daily intakes of different metals within the framework of defined scenarios are compared with respective chronic oral reference doses to assess the existent hazards. Details for exposure assessment and hazard analysis are fully described within the literature [20].

## Results

Total concentration of mentioned metals in soil samples collected from the vicinity of Mubarakeh steel complex are shown in Table 4.

**Table 4:** Bulk concentration of metals (mg/kg, dry mass) in soil samples within the study area

Station	Co	Mn	As	Ni	Pb	Cr	Cd	Cu	Ba	Mo	Zn	V	Fe %	Al %
S1	1.80	391.98	6.39	49.63	6.84	53.66	0.98	27.46	94.31	0.69	70.85	25.27	1.89	0.10
S2	2.31	455.58	6.67	47.35	2.84	38.22	0.92	33.08	95.64	0.45	67.03	25.64	1.37	0.10
S3	5.88	419.01	6.28	45.91	7.58	40.77	0.84	72.39	68.33	0.69	87.91	31.49	1.45	0.11
S4	3.90	501.45	8.56	64.20	20.84	47.45	4.34	70.18	98.77	0.35	95.72	27.34	1.61	0.11
S5	3.66	603.02	9.99	80.00	8.62	59.70	1.24	59.07	98.87	0.49	102.81	25.78	2.19	0.12
S6	4.90	560.10	8.43	65.06	8.42	47.19	1.08	44.01	87.62	0.47	77.03	24.55	1.64	0.13
S7	2.37	465.56	7.30	65.21	10.61	45.64	1.14	35.89	100.81	0.61	80.71	22.70	1.85	0.13
S8	2.43	445.69	8.64	66.53	7.55	43.88	0.98	31.68	71.48	0.76	73.95	23.26	1.58	0.12
S9	2.18	341.01	7.34	59.28	6.41	46.01	4.77	38.51	88.40	0.24	68.30	19.04	1.43	0.12
S10	3.03	421.85	7.14	59.58	7.93	42.74	1.01	29.38	73.71	0.28	70.09	18.26	1.46	0.10
S11	1.73	414.25	8.42	58.69	10.77	42.94	1.06	36.40	93.12	0.41	87.59	21.18	1.50	0.12
S12	1.88	466.20	8.88	69.48	9.55	47.47	1.10	32.02	75.65	0.72	79.21	20.92	1.67	0.13
S13	1.00	398.80	9.01	70.65	8.11	47.77	1.07	31.34	68.14	0.64	81.15	16.91	1.70	0.13
S14	2.04	341.36	11.20	55.94	8.49	37.43	0.91	39.63	68.66	0.31	74.13	20.28	1.34	0.11
Max	5.88	603.02	11.20	80.00	20.84	59.70	4.77	72.39	100.81	0.76	102.81	31.49	2.19	0.13
Min	1.00	341.01	6.28	45.91	2.84	37.43	0.84	27.46	68.14	0.24	67.03	16.91	1.34	0.10
SD	1.35	73.93	1.41	9.52	3.94	5.82	1.29	14.85	12.83	0.18	10.58	3.96	0.23	0.01
Mean	2.79	444.70	8.16	61.25	8.90	45.78	1.53	41.50	84.54	0.51	79.75	23.04	1.62	0.12
Shale values	19	850	13	68	20	90	0.3	45	498	2.6	95	130	4.70	8.2

In comparison with average shale values, all metals show lower concentrations except for Cd. Average cadmium concentration within the study area is more than five times higher than that of shale which indicate an intensified enrichment. On the other hand, although the concentration of other metals is lower than that of shale, such condition should not be considered as a low-risk and safe one. Potential mobility of existing metallic concentration in soil should be regarded before any judgment.

The results of chemical partitioning for metals are demonstrated in Figs. 3, 4, 5, 6, 7, 8, 9, 10, 11, 12, 13 and 14.

**Fig. 3:**
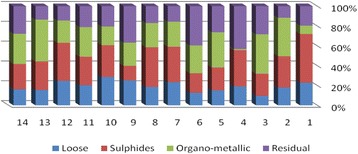
Chemical partitioning of Cobalt in soil samples

**Fig. 4:**
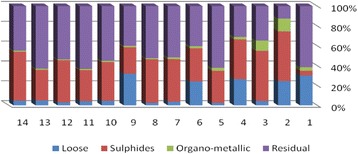
Chemical partitioning of Manganese in soil samples

**Fig. 5:**
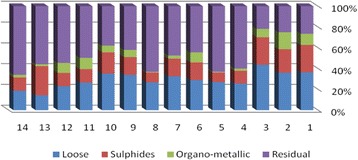
Chemical partitioning of Arsenic in soil samples

**Fig. 6:**
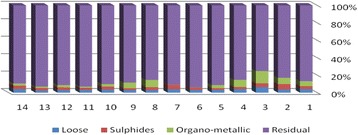
Chemical partitioning of Nickel in soil samples

**Fig. 7:**
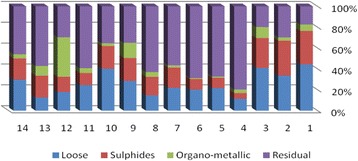
Chemical partitioning of Lead in soil samples

**Fig. 8:**
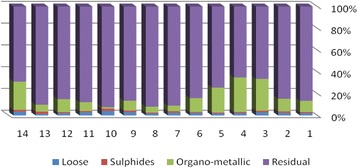
Chemical partitioning of Chromium in soil samples

**Fig. 9:**
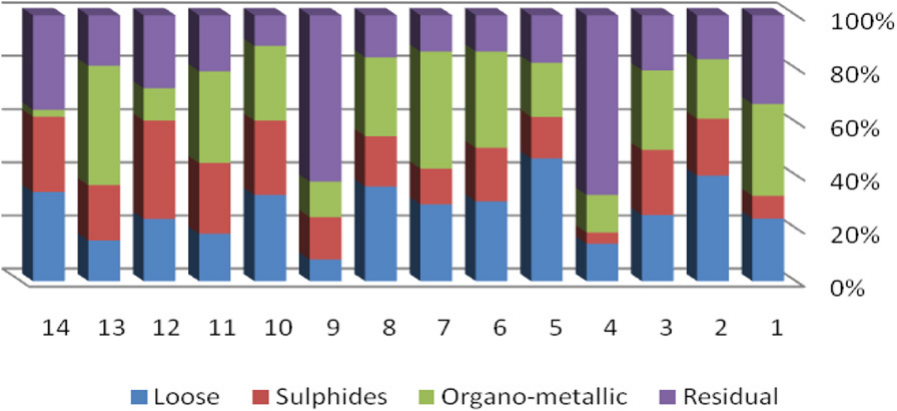
Chemical partitioning of Cadmium in soil samples

**Fig. 10:**
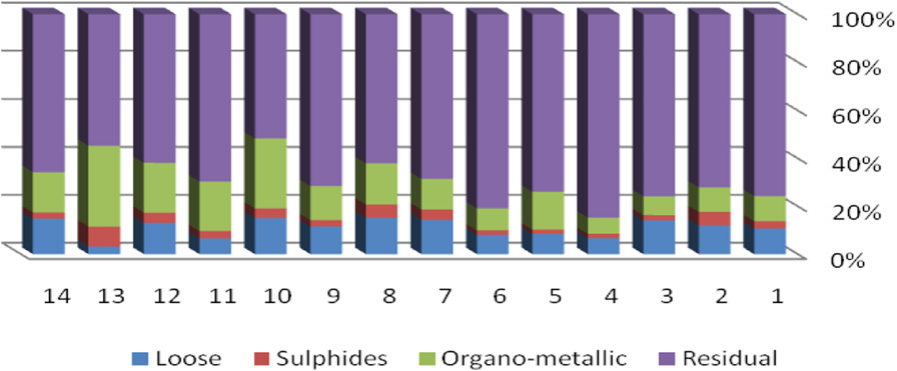
Chemical partitioning of Copper in soil samples

**Fig. 11:**
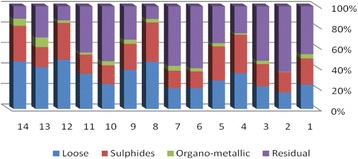
Chemical partitioning of Barium in soil samples

**Fig. 12:**
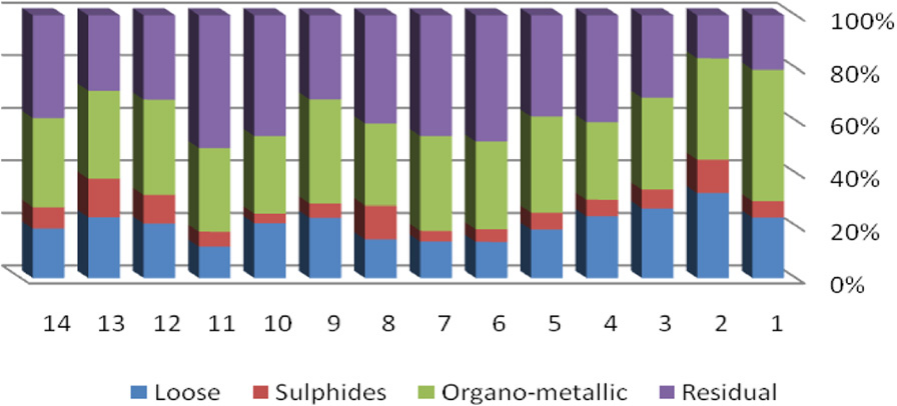
Chemical partitioning of Molybdenum in soil samples

**Fig. 13:**
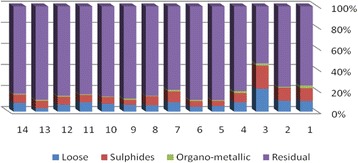
Chemical partitioning of Zinc in soil samples

**Fig. 14:**
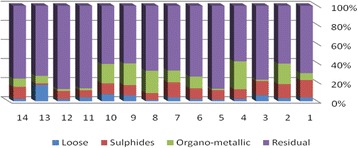
Chemical partitioning of Vanadium in soil samples

The percent of metals concentration bound to different phases are grouped in a descending order as follows:

Loose ions :

Ba (31.53) > As (29.17) > Cd (26.73) > Pb (25.82) > Mo (20.37) > Co (19.21) > Mn (12.23) > Cu (10.98) > Zn (8.27) > V (4.68) > Ni (3.53) > Cr (2.48)

Sulfide ions :

Mn (36.97) > Co (30.51) > Ba (25.88) > Cd (20.26) > Pb (19.22) > As (17.48) > V (10.92) > Zn (8.96) > Mo (7.74) > Cu (3.67) > Ni (3.36) > Cr (1.25)

Organic ions :

Mo (35.39) > Co (26.30) > Cd (26.10) > Cu (16.12) > Cr (13.72) > V (12.33) > Pb (7.48) > As (6.38) > Ni (4.65) > Ba (3.75) > Mn (3.18) > Zn (1.15)

Residual ions :

Ni (88.41) > Cr (82.55) > Zn (81.61) > V (72.07) > Cu (69.23) > Mn (47.62) > Pb (47.48) > As (46.97) > Ba (38.84) > Mo (36.50) > Cd (26.91) Co (23.98)

In order to have an overall estimate of metals mobility potential, the results of the sum of loose, sulfide and organic ions are grouped as the anthropogenic portion of metals are shown in Table 5.

**Table 5:** Percentile of anthropogenic portion in bulk metallic concentration of soil samples

Station	Anthropogenic portion of metallic pollution (%)													
	Al	Co	Mn	As	Ni	Pb	Cr	Cd	Cu	Ba	Fe	Mo	Zn	V
1	11.54	72.31	34.58	66.14	11.80	74.41	12.01	59.90	21.86	47.99	0.78	71.34	22.27	26.47
2	17.93	79.47	78.68	67.46	15.31	63.25	13.78	75.13	25.09	32.48	0.77	75.19	21.15	35.46
3	5.74	64.45	58.75	70.40	22.32	72.17	30.21	71.34	21.77	42.31	2.29	61.82	41.24	20.26
4	11.87	51.15	62.07	35.99	13.22	18.23	31.37	29.26	13.76	66.41	0.40	53.43	17.48	37.59
5	4.12	66.29	34.02	33.28	7.82	29.40	22.99	73.88	23.44	57.60	0.21	55.32	9.83	11.79
6	5.04	54.36	53.67	50.04	5.26	28.05	14.31	77.69	17.19	35.70	0.32	46.77	10.00	23.14
7	5.44	75.91	43.47	47.32	8.14	39.16	8.00	77.62	28.30	37.74	0.73	48.67	18.58	28.94
8	7.75	74.68	42.77	33.19	13.19	33.31	7.20	75.75	34.16	78.91	0.47	53.02	13.56	28.67
9	7.68	56.89	53.93	52.16	10.37	58.51	12.13	33.73	25.59	60.73	0.32	61.23	11.07	35.70
10	3.64	71.54	40.39	55.79	8.89	58.11	6.77	79.60	43.46	41.90	0.75	48.65	12.85	35.08
11	2.03	71.01	33.00	45.29	6.24	36.63	10.91	71.00	27.29	49.42	0.33	44.50	14.76	12.15
12	6.64	76.99	41.71	40.87	7.80	63.31	13.47	65.22	34.30	77.72	0.71	61.16	13.48	11.47
13	9.94	77.84	33.28	39.72	6.18	38.57	8.84	72.83	40.69	62.64	0.19	64.24	10.13	23.88
14	3.04	64.97	49.61	30.54	9.44	48.62	27.87	58.02	30.79	79.14	0.51	54.80	15.25	21.32
Max	17.93	79.47	78.68	70.40	22.32	74.41	31.37	79.60	43.46	79.14	2.29	75.19	41.24	37.59
Min	2.03	51.15	33.00	30.54	5.26	18.23	6.77	29.26	13.76	32.48	0.19	44.50	9.83	11.47
SD	4.30	9.08	13.23	13.31	4.54	17.75	8.66	15.91	8.40	16.38	0.52	9.13	8.16	9.11
Mean	7.31	68.42	47.14	47.73	10.43	47.27	15.70	65.78	27.69	55.05	0.63	57.15	16.55	25.14

As was explained in methodology, IPOLL, Bc, and Lp refers to pollution intensity, bulk concentration, and lithogenous portion, respectively. Average indices values for each metal in different stations are shown in Table 6.

**Table 6:** Mean values of various pollution indices in soil samples

Index	Al	Co	Mn	As	Ni	Pb	Cr	Cd	Cu	Ba	Fe	Mo	Zn	V
Igeo	−6.75	−3.50	−1.54	−1.28	−0.75	−2.00	−0.36	1.94	−0.57	−3.10	−2.14	−3.04	−1.19	−3.10
Ipoll	0.11	1.72	0.97	0.99	0.16	1.01	0.25	1.66	0.48	1.26	0.01	1.26	0.27	0.43
EF	0.04	0.44	1.45	17.05	2.38	2.03	1.42	48.67	2.60	0.64	1.00	1.04	3.31	0.45

The result of metal concentration in agricultural crops (wheat and rice), are shown in Tables 7 and 8.

**Table 7:** concentration of metals in different rice samples from the study area (mg/kg dry weight)

ID	Al	Co	Mn	As	Ni	Pb	Cr	Cd	Cu	Ba	Fe	Mo	Zn	V
Rice 1	463.15	0.47	16.86	1.79	6.61	1.04	10.41	0.29	3.88	4.43	9.74	2.64	28.72	2.46
Rice 2	214.05	0.44	15.91	0.62	6.63	0.86	8.46	0.32	6.06	4.24	16.37	1.61	32.32	3.35
Rice 3	211.00	0.37	14.81	1.24	5.03	1.22	7.16	0.40	4.01	4.95	8.89	1.77	22.84	3.01
Rice 4	227.10	0.44	15.42	1.14	9.94	1.51	9.25	0.34	4.57	4.52	9.63	0.85	31.79	3.96
Rice 5	480.00	0.51	16.32	1.26	7.17	1.33	9.81	0.29	5.02	5.42	10.31	1.74	29.79	3.87
Rice 6	250.25	0.49	18.64	1.57	6.07	0.64	9.03	0.35	5.70	4.13	8.26	1.52	31.77	2.99
Rice 7	528.10	0.47	13.11	1.52	6.83	3.34	11.83	0.35	3.80	4.71	21.44	1.59	35.45	3.27
Rice 8	572.25	0.42	22.92	1.47	5.93	0.42	9.91	0.26	8.21	5.58	31.63	1.89	32.93	6.46
Mean	368.24	0.45	16.75	1.33	6.78	1.30	9.48	0.33	5.16	4.75	14.53	1.70	30.70	3.67
St.Dev	156.30	0.05	2.96	0.35	1.44	0.90	1.38	0.05	1.49	0.53	8.28	0.49	3.76	1.23

**Table 8:** concentration of metals in different wheat samples from the study area (mg/kg dry weight)

ID	Al	Co	Mn	As	Ni	Pb	Cr	Cd	Cu	Ba	Fe	Mo	Zn	V
Wheat 1	294.85	0.46	52.17	1.11	5.89	1.23	8.55	1.26	9.23	5.26	11.29	1.93	38.55	6.61
Wheat 2	226.75	0.44	14.84	1.00	6.39	0.34	8.09	0.25	7.80	6.12	16.08	1.42	30.96	6.86
Wheat 3	329.35	0.53	53.54	1.95	6.87	1.09	9.37	0.25	10.67	7.34	13.81	1.81	19.85	8.91
Wheat 4	394.35	0.44	48.11	0.94	4.86	0.91	8.33	0.33	7.79	5.46	12.76	1.76	49.51	6.67
Wheat 5	493.70	0.46	53.86	3.11	6.77	1.07	10.42	1.16	7.90	8.00	14.38	1.55	28.56	7.29
Wheat 6	623.70	0.38	26.92	1.26	5.79	1.02	10.12	0.33	6.08	5.25	105.74	1.92	27.49	4.17
Wheat 7	344.15	0.47	61.94	1.13	6.92	0.49	9.80	0.27	11.38	7.40	17.49	1.86	49.32	7.92
Wheat 8	173.45	0.49	62.96	1.79	6.10	0.83	7.17	0.28	10.36	7.12	33.50	1.61	39.40	8.91
Wheat 9	148.70	0.54	70.96	1.98	10.18	1.92	7.51	0.38	11.50	7.87	18.96	1.39	41.68	8.84
Wheat 10	480.65	0.45	43.56	0.51	5.36	0.41	8.99	0.25	6.41	9.11	24.76	1.69	26.33	6.71
Wheat 11	230.25	0.71	83.25	2.29	7.48	1.23	7.76	0.25	12.81	10.22	27.59	1.47	54.04	11.05
Wheat 12	82.10	0.47	46.19	2.45	7.72	2.80	6.72	0.30	13.13	6.28	14.94	1.38	53.83	6.84
Wheat 13	185.30	0.48	63.82	1.94	7.35	1.19	10.22	0.29	8.42	12.26	10.21	1.86	46.50	8.21
Wheat 14	398.95	0.47	32.56	1.34	6.46	0.69	9.58	0.25	6.32	6.95	8.36	1.72	27.70	4.48
Wheat 15	499.10	0.43	54.17	1.15	5.87	0.43	9.05	0.26	8.14	5.13	20.83	2.02	41.91	8.11
Mean	327.02	0.48	51.26	1.60	6.67	1.04	8.78	0.41	9.20	7.32	23.38	1.69	38.37	7.44
St.Dev	154.67	0.07	17.33	0.69	1.26	0.64	1.16	0.33	2.32	2.00	23.79	0.21	10.99	1.74

According to defined scenarios in part of material and methods, respective chronic oral reference doses to assess the existent hazards are shown in Tables 9 and 10. Details for exposure assessment and hazard analysis are fully described within the literature [20].

**Table 9:** Chronic daily intakes of metals through different scenarios due to rice consumption

ID	Mean concentration ppb	Scenario 1 mg/kg-day	Scenario 2 mg/kg-day	Scenario 3 mg/kg-day	Scenario 4 mg/kg-day	Scenario 5 mg/kg-day	Scenario 6 mg/kg-day	Oral RfD mg/kg-day
Al	368.24	5.26E-01	1.32E + 00	2.10E + 00	2.45E + 00	6.14E + 00	9.82E + 00	1.00E + 00
Co	0.45	6.43E-04	1.61E-03	2.57E-03	3.00E-03	7.50E-03	1.20E-02	3.00E-04
Mn	16.75	2.39E-02	5.98E-02	9.57E-02	1.12E-01	2.79E-01	4.47E-01	1.40E-01
As	1.33	1.90E-03	4.75E-03	7.60E-03	8.87E-03	2.22E-02	3.55E-02	3.00E-04
Ni	6.78	9.69E-03	2.42E-02	3.87E-02	4.52E-02	1.13E-01	1.81E-01	2.00E-02
Cr	9.48	1.35E-02	3.39E-02	5.42E-02	6.32E-02	1.58E-01	2.53E-01	3.00E-03
Cd	0.33	4.71E-04	1.18E-03	1.89E-03	2.20E-03	5.50E-03	8.80E-03	1.00E-03
Cu	5.16	7.37E-03	1.84E-02	2.95E-02	3.44E-02	8.60E-02	1.38E-01	4.00E-02
Ba	4.75	6.79E-03	1.70E-02	2.71E-02	3.17E-02	7.92E-02	1.27E-01	2.00E-01
Fe	14.53	2.08E-02	5.19E-02	8.30E-02	9.69E-02	2.42E-01	3.87E-01	7.00E-01
Mo	1.7	2.43E-03	6.07E-03	9.71E-03	1.13E-02	2.83E-02	4.53E-02	5.00E-03
Zn	30.7	4.39E-02	1.10E-01	1.75E-01	2.05E-01	5.12E-01	8.19E-01	3.00E-01
V	3.67	5.24E-03	1.31E-02	2.10E-02	2.45E-02	6.12E-02	9.79E-02	7.00E-05

**Table 10:** Chronic daily intakes of metals through different scenarios due to wheat consumption

ID	Mean concentration ppb	Scenario 1 mg/kg-day	Scenario 2 mg/kg-day	Scenario 3 mg/kg-day	Scenario 4 mg/kg-day	Scenario 5 mg/kg-day	Scenario 6 mg/kg-day	Oral RfD mg/kg-day
Al	327.02	7.01E-01	1.40E + 00	2.10E + 00	3.27E + 00	6.54E + 00	9.81E + 00	1.00E + 00
Co	0.48	1.03E-03	2.06E-03	3.09E-03	4.80E-03	9.60E-03	1.44E-02	3.00E-04
Mn	51.26	1.10E-01	2.20E-01	3.30E-01	5.13E-01	1.03E + 00	1.54E + 00	1.40E-01
As	1.6	3.43E-03	6.86E-03	1.03E-02	1.60E-02	3.20E-02	4.80E-02	3.00E-04
Ni	6.67	1.43E-02	2.86E-02	4.29E-02	6.67E-02	1.33E-01	2.00E-01	2.00E-02
Cr	8.78	1.88E-02	3.76E-02	5.64E-02	8.78E-02	1.76E-01	2.63E-01	3.00E-03
Cd	0.41	8.79E-04	1.76E-03	2.64E-03	4.10E-03	8.20E-03	1.23E-02	1.00E-03
Cu	9.2	1.97E-02	3.94E-02	5.91E-02	9.20E-02	1.84E-01	2.76E-01	4.00E-02
Ba	7.32	1.57E-02	3.14E-02	4.71E-02	7.32E-02	1.46E-01	2.20E-01	2.00E-01
Fe	23.38	5.01E-02	1.00E-01	1.50E-01	2.34E-01	4.68E-01	7.01E-01	7.00E-01
Mo	1.69	3.62E-03	7.24E-03	1.09E-02	1.69E-02	3.38E-02	5.07E-02	5.00E-03
Zn	38.37	8.22E-02	1.64E-01	2.47E-01	3.84E-01	7.67E-01	1.15E + 00	3.00E-01
V	7.44	1.59E-02	3.19E-02	4.78E-02	7.44E-02	1.49E-01	2.23E-01	7.00E-05

As it is seen except for Fe, Ba, Cu and Zn, all metals intakes in different scenarios are considered to be hazardous while their CDI values are much more than the respective oral reference doses.

## Discussion

Evaluating the metallic pollution of soil and the potential mobility of metal species in the vicinity of Mubarakeh steel complex is considered in this study. Fourteen soil samples were collected and analyzed in summer 2012.

Bioavailability and health risks linked to toxic metals are tightly related to the mobility of metal species. aqueous organisms and plants are more vulnerable towards free ions and labile complexes [3, 21–23]. Accordingly, it may be concluded from the chemical partitioning data that there are significant differences in the distribution of the metals studied. Cobalt, Cd, As, Mo and Pb are introduced as the most risky metals since they present the highest percentages in loose, sulfide and organic fractions and the lowest in residual form. Accordingly, exchange of mentioned metals may be expected between the soil and water column.

Sum of loose, sulfide and organic phases in bulk metallic concentration is considered as manipulated contamination. The higher the anthropogenic portion of a metal is, the higher the mobility risk would be occurred. Generally, the mean anthropogenic portions of the metals, as a percent of their bulk concentrations, obey the following pattern:Co > Cd > Mo > Ba > As > Pb > Mn > Cu > V > Zn > Cr > Ni.

## Conclusions

Considering index approach, Igeo deals with bulk concentration and is constructed upon the principle of comparison with shale values. As it is expected, only cadmium is detected to be in a moderately contaminated status regarding this index while other metals declare an unpolluted condition. Index of pollution pays more attention to mobility potential of metals and accordingly detects metals Co, Mn, As, Pb, Cd, Ba and Mo to be in a moderately contaminated level. On the other hand, enrichment factor which compares the bulk concentrations with earth crust values declares all toxic metals except for Co, Ba and V to be enriched.

Considering human health hazard assessment, all toxic metals intakes in different scenarios are considered to be hazardous. The status is much more severe for scenarios dealt with children eating more portions. It must be noted that current hazard levels are calculated as single exposures and for more realistic conditions cumulative exposures should be taken into consideration. In other words, a variety of ingredients (containing rice, wheat, water, etc.) exists in diurnal diet of residents within the study area and the overall hazard would exceed the above-mentioned levels.
